# Calves shedding *Mycobacterium avium* subspecies *paratuberculosis* are common on infected dairy farms

**DOI:** 10.1186/s13567-015-0192-1

**Published:** 2015-06-19

**Authors:** Robert Wolf, Karin Orsel, Jeroen De Buck, Herman Wildrik Barkema

**Affiliations:** Department of Production Animal Health, Faculty of Veterinary Medicine, University of Calgary, Alberta, Canada

## Abstract

*Mycobacterium avium* subspecies *paratuberculosis* (MAP) causes Johne’s disease, a chronic progressive enteritis. It is generally assumed that calves rarely shed MAP bacteria and that calf-to-calf transmission is of minor importance. The objectives were 1) to estimate the prevalence of MAP-shedding young stock in MAP-infected dairy herds, and identify predictors for test-positive young stock; and 2) to estimate proportions of MAP-contaminated young stock group housing pens and air spaces, and furthermore, identify predictors for test-positive pens. Fecal samples were collected from 2606 young stock on 18 MAP-infected dairy farms. Environmental fecal samples were collected from all group-housing pens and dust samples were collected from all barns. All individual samples were analysed using IS900 and F57 qPCR; fecal samples positive by either PCR and all environmental and dust samples were cultured. Overall, 8.1, 1.2 and 2.0% of cattle were positive on IS900 qPCR, F57 qPCR and bacterial culture, respectively. Young stock housed on farms with culture-positive environmental samples collected from adult cow housing and manure storage had higher odds of testing IS900 qPCR-positive than young stock housed on farms with only negative environmental samples. Furthermore, 14% of collected environmental samples, but no dust samples, were test-positive. Age of cattle in the pen was a significant predictor for environmental sample results. Young stock excreted MAP bacteria in their feces which provided strong evidence for calves as sources of within-herd transmission of MAP on dairy farms known to be infected with this organism.

**Electronic supplementary material:**

The online version of this article (doi:10.1186/s13567-015-0192-1) contains supplementary material, which is available to authorized users.

## Introduction

*Mycobacterium avium* subspecies *paratuberculosis* (MAP) causes Johne’s disease (JD), a chronic progressive enteritis in ruminants [1]. This disease is common in dairy herds and causes substantial economic losses through decreased milk production and slaughter value, and increased risk of premature culling [2,3]. In Alberta, Canada > 50% of herds and 18% of dairy cows are infected with MAP [4,5].

Susceptibility to MAP infection is highest in young animals [1,6]. Cattle get infected in utero or through ingestion of infectious colostrum, milk, or feces. After infection, the incubation period is prolonged (typically 2 to 5 years, but up to 10 years). During incubation, frequency of shedding as well as fecal concentrations of MAP bacteria increase [7,8]. These assumptions regarding susceptibility and bacterial shedding have been implemented into JD simulation models, which are frequently used to design control programs [9-11]. Consequently, control programs focus on interrupting direct and indirect contact between likely shedding adult cows and highly susceptible calves [12-14]. However, in two recent infection trials, a high proportion of calves shed MAP soon after experimental challenge, with some calves shedding as early as two months after exposure [15,16]. Field studies provide inconsistent results, with two studies identifying MAP shedding young stock on infected dairy farms [17,18], but one other study reporting no evidence for MAP-shedding calves [19]. There are similar inconsistencies with regards to studies estimating relevance of MAP transmission between young stock. For example, whereas one transmission trial reported evidence for transmission between young stock [20], another transmission experiment did not detect any [15], and two simulation studies regarded transmission between young stock as irrelevant for the spread of MAP [21,22]. Accurate knowledge regarding importance of transmission routes is essential to design future control programs; the first step is to reduce uncertainty with respect to occurrence and prevalence of MAP shedding young stock in infected herds. There is, therefore, a need for an observational study, conducted on many MAP-infected dairy herds, estimating proportions of MAP-shedding young stock in various age groups. Furthermore, detection of MAP in group housing pens would provide strong evidence for MAP contaminated environment as a risk factor for MAP infection in young stock.

The objectives were: 1) to estimate prevalence of MAP shedding young stock in MAP-infected dairy herds, and identify predictors for test-positive young stock; and 2) to estimate proportions of MAP-contaminated young stock group housing and air spaces, and identify predictors for test-positive pens.

## Materials and methods

### Herds

Based on the average herd size of 145 cows in Alberta [23], it was expected that 10 cattle within an age range of three months would be available for sampling at any point in time in each herd, which would result in an overall total of 180 cattle in this age group, a sample size sufficient to detect a minimum prevalence of 2% [24]. Farms were selected among 360 farms voluntarily participating in the Alberta Johne’s Disease Initiative (AJDI, >60% of Alberta dairy farms participate). Eligible producers had ≥ 1 MAP culture-positive environmental sample during one of the previous AJDI sampling events [3], and were clients of 1 of 4 veterinary clinics with a major focus on dairy. A total of 20 randomly selected farms needed to be approached to achieve the target sample size of 18 participants. Reasons for refusal of participation were lack of interest in one case and fear of disease introduction by sampling personnel in the other case.

### Sample collection, shipping and processing

Samples were collected between May 2013 and January 2014. Herd size, history of observed clinical JD, and number of MAP-positive environmental samples collected from adult cow housing and manure storage were available through AJDI records. Fecal samples were collected from the rectum (using lubricated gloves) of all female dairy cattle before first calving, and all male cattle <30 months of age. The presence of watery diarrhea was recorded. Sample collection was not conducted if animals were on pasture where they could not be easily restrained (all young stock > 6 months of age from one farm).

A single environmental manure sample was collected from each of the calf group-housing pens. These samples were composed of four well-mixed sub-samples, preferably collected from alleys, or around waterers [4]. If pens did not have these areas, samples were collected from bedding packs or exercise areas. Samples were not collected if pens were occupied by only one animal. Settled dust was collected in barns and sheds (one sample from each barn) using a commercially available dust swipe (12 × 12 cm) wiping a length ~0.5 m long in areas with settled dust and out of reach for cattle [25]. Environmental manure samples and dust samples were not collected if sample collection criteria were not met, e.g., groups maintained on pasture.

Samples were transported to University of Calgary on the day of collection and stored at 4 °C (maximum of 21 days). The decision to store samples at 4 °C instead of −20 or −70 °C was motivated by available freezer space and expected losses in numbers of viable MAP bacteria during freezing and thawing [26]. Laboratory procedures were as described [25,27]. In short, all individual fecal samples were processed using IS900 and F57 qPCR; a MagMAX total nucleic acid isolation kit (Applied Biosystems, Carlsbad, CA, USA) was used for DNA extraction. 40 PCR cycles were completed and samples were considered positive if a signal was detected before 37 cycles. However, all samples with a signal within 40 cycles on at least one of the two PCR methods were cultured (if enough feces had been collected). Furthermore, all PCR-positive samples were cultured from 13 farms, but only a subset of PCR-positive samples were cultured from the first five farms.

A standardized TREK ESP culture protocol with a three-day decontamination, followed by a 48-day incubation period and confirmation using conventional IS900 PCR, was used [27]. All environmental samples were cultured using the same protocol as for individual fecal samples. Dust samples were processed with a slightly modified culture protocol, as described [25]. The laboratory was USDA certified to conduct all required procedures. Furthermore, positive as well as negative culture and PCR controls were added to any processing batch, aiming to detect cross contamination as well as laboratory protocol failures.

### Statistical analyses

Analyses were conducted using STATA Version 11 (Statacorp, College Station, TX, USA). While the prevalence of MAP shedding calves using IS900 and F57 qPCR was determined using results from all 18 herds, culture prevalence was estimated using samples from 13 herds where a serial testing scheme was performed.

Chi-square tests on contingency tables were used to compare herd size, history of clinical JD, and environmental sample results between study participants and non-participants, within the population of farms participating in the AJDI.

Chi-square tests on contingency tables were used to screen data for associations between test positives (qPCR case definition: ct-value < 37 cycles, culture case definition: ct-value < 40 cycles on at least one of the two PCR methods and subsequently positive on culture) and animal characteristics, including age (<3 months, 3 – 6 months, 6 months – 1 year, 1 year – 2 years, or > 2 years), diarrhea (yes/no), and number of culture-positive adult cow environmental samples (0, 1 – 3, or 4 – 6 positives out of 6 collected samples) as an indicator for the adult cow within-herd prevalence [28]. Following initial univariate analyses, multivariable models were built which adjusted for confounding and clustering within farms [29]. Three models were built using either the IS900 qPCR result, the F57 qPCR result, or the culture result as the outcome variable. Farm and pen were included as random effects. Although IS900 and F57 models used data from all 18 herds, the culture model used data from the 13 herds with a serial-testing scheme. Stepwise backward selection with a *P* ≤ 0.05 cutoff value was used for variable exclusion in logistic models. A predictor change of 20% was used as a threshold to identify confounding. Coefficients and odds ratios (OR) were cluster-specific. Therefore, the presented OR described the median OR for shedding MAP among all pen-specific ORs [29].

Three separate models were constructed to identify predictors for MAP-contaminated group housing pens. The environmental sample culture result was used as the outcome and the percentage MAP shedding calves was included by using either percentage of 1) IS900, 2) F57, or 3) culture-positive cattle in a pen (0, 1 – 20%, or > 20%) as predictor. Median age of cattle in a pen (<3 months, 3 – 6 months, 6 months – 1 year, 1 – 2 years, or ≥ 2 years), number of cattle in a pen (1, 2 – 9, or > 9), and number of culture-positive adult cow environmental samples during the last annual test event (0, 1 – 3, or 4 – 6 positives out of 6 collected samples), were considered as additional predictors in all three models. Farm was included as a random effect.

## Results

The 18 participating farms had a mean herd size of 156 cows. Whereas 56% of the farms participating in the study had observed clinical JD on their farm, 29% of non-participants had also observed clinical JD (*P* = 0.03; Table 1). Although 11% of the farms participating in the study tested negative on all environmental samples, 55% of non-participants tested negative on all environmental samples (*P* < 0.01).

**Table 1:** **Herd characteristics for study farms and farms participating in the Alberta Johne’s Disease Initiative (AJDI,** ***n*** **(%))**

	**Study participants (** ***n*** **= 18)**	**Other AJDI farms (** ***n*** **= 342)**
Herd size		0.69^1^
<50	0 (−)	9 (3)
50 – 99	2 (11)	102 (30)
100 - 149	8 (44)	130 (38)
150 – 199	3 (17)	48 (14)
>199	4 (22)	53 (15)
History of clinical Johne’s disease		0.03^1^
JD has been observed	10 (56)	98 (29)
Don’t know	4 (22)	74 (22)
JD has never been observed	4 (22)	170 (49)
Positive environmental samples		< 0.01^1^
0 positives	2 (11)^2^	188 (55)
1-3 positives	9 (50)	84 (25)
4-6 positives	7 (39)	70 (20)

^1^ *P*-value based on Chi-square test on contingency table.

^2^These 2 herds had no MAP culture-positive environmental samples at the last testing event, but had positive environmental samples in 1 of the 2 previous samplings.

A total of 2606 young stock were sampled in 18 herds. There were 210 young stock positive on IS900 qPCR, 32 were positive on F57 qPCR, and 10 calves were positive on both pPCR methods. This resulted in a prevalence of 8.1% (95% CI: 7.0 – 9.1%) based on IS900 and 1.2% (95% CI: 0.8 – 1.7%) based on F57 qPCR.

**Figure 1:**
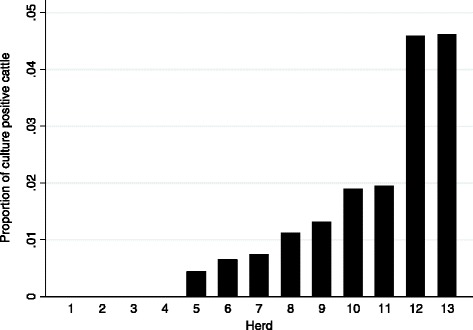
**Proportions of** ***Mycobacterium avium*** **subsp**. ***paratuberculosis*** **culture-positive cattle.** Culture was conducted on 13 farms (*n* = 1741); 4 farms had only negative samples.

There were 1741 young stock sampled in the 13 herds where serial testing was performed (Additional file 1). Of the 1741 young stock, 192 (11.0%) had a ct-value < 40 on IS900 qPCR and 44 (2.5%) had a ct-value < 40 on F57 qPCR. Furthermore, 216 (12.4%) young stock had a ct-value < 40 in any of the two qPCR methods and were consequently eligible for culture. Seven PCR-positive samples included insufficient amounts of fecal material for MAP culture and were removed from the analysis. 34 PCR-positives were also culture-positive, resulting in a MAP shedding prevalence of 2.0% (95% CI: 1.3 – 2.6%). On those 13 farms, within-herd culture prevalence ranged from 0 to 4.6% (Figure 1).

**Figure 2:**
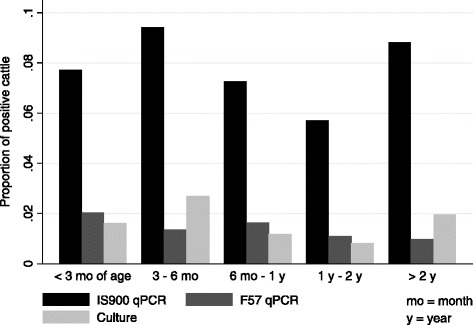
**Age-specific proportions of cattle excreting** ***Mycobacterium avium*** **subsp**. ***paratuberculosis*** **in their feces.** IS900 and F57 qPCR and culture were conducted on 13 farms (*n* = 1741; mo = month, y = year).

Positive cattle were present in all age groups (Figure 2), and number of IS900-positive cattle was positively associated with number of positive environmental samples collected in adult cow housing and manure storage areas (*P* < 0.01). In the final logistic regression model, young stock housed on farms with 1 – 3 positive environmental samples collected from adult cow housing and manure storage had 11.5 times the odds (95% CI: 1.3 – 100.0), and young stock housed on farms with 4 – 6 positive environmental samples had 9.7 times the odds (95% CI: 1.1 – 86.0) of testing IS900 qPCR-positive, respectively, than young stock housed on farms with only negative environmental samples in their last sampling event (Table 2). None of the independent variables significantly predicted F57 or culture results as outcomes in separate logistic regression models.

**Table 2:** **Predictors for** ***Mycobacterium avium*** **subsp.** ***paratuberculosis*** **IS900 qPCR results on individual fecal samples (** ***n*** **= 2606)**

	***n*** **(% pos)** ^**1**^	**OR**	**95% CI**	***P*** **-value**
Intercept		0.01	0.00 - 0.05	< 0.01
Pos. env. samples^2^				0.09
0	196 (1)	Reference		
1 – 3	1,276 (10)	11.53	1.33 – 100.04	0.03
4 – 6	1,134 (7)	9.70	1.09 – 86.30	0.04
Random effects		Var. (SE)		% Var.
Herd		0.71 (0.33)		16
Pen		0.50 (0.22)		11
Animal		-		73

Descriptive statistics and final multilevel logistic regression model.

^1^Sample size (% IS900 PCR-positive samples).

^2^Positive environmental samples: stratification according to the number of culture-positive environmental samples collected from adult cow housing and manure storage areas.

Environmental samples were collected from 139 (88%) of 155 group-housing pens. Of these, 20 (14%) samples were MAP culture-positive, and 9 (50%) of the 18 farms had positive environmental samples (within-herd environmental sample prevalence ranged from 0 to 43%; Additional file 1). In the final logistic regression model, pens with cattle in the age group between 6 months and 1 year had 10.5 times the odds (1.0 – 116.9) for being environmental culture-positive compared to pens with cattle < 3 months of age (Table 3). In separate models, neither F57 nor culture prevalence were significant predictors for environmental sample results. Finally, none of the 41 collected dust samples were MAP culture-positive.

**Table 3:** **Predictors for** ***Mycobacterium avium*** **subsp.** ***paratuberculosis*** **environmental sample culture results (** ***n*** **= 139)**

	***n*** **(% pos)** ^**1**^	**Odds/OR**	**95% CI**	***P*** **-value**
Intercept		0.01	0.00 – 0.16	<0.01
Age group				0.39
<3 months	31 (3)	Reference		
3 – 6 months	39 (13)	4.67	0.46 – 47.65	0.19
6 months – 1 year	25 (24)	10.52	1.01 - 116.87	0.05
1 – 2 years	38 (18)	7.56	0.74 – 76.91	0.09
>2 years	6 (17)	9.57	0.38 – 240.56	0.17
IS900 within-pen prevalence^2^				0.92
0	75 (12)	Reference		
0.01 – 0.19	42 (17)	0.94	0.27 – 3.26	0.93
>0.2	22 (18)	1.32	0.25 – 6.93	0.74
Random effects		Var. (SE)		% Var.
Herd		0.70 (0.89)		17
Pen		-		83

Descriptive statistics and final multilevel logistic regression model.

^1^Sample size (% culture-positive samples).

^2^Parameter included in the model because of evidence for an association in the descriptive statistics and biological plausibility.

## Discussion

Calves and young stock that excreted MAP in their feces were present in all age groups. A high proportion of group housing pens was contaminated with MAP; positive test results were associated with age of cattle and prevalence of MAP-shedding animals in the pen. However, all analysed dust samples were MAP-negative, suggesting a minimal role of dust as a vehicle for transmission of MAP on dairy farms, particularly since young stock and adult cattle are often housed separately.

Overall, 2.0% of young stock were culture-positive, confirming results of two other studies that reported 3 and 2% MAP culture-positive young stock, respectively [17,18]. Although prevalence estimates in the present study were comparable to those of the two other studies, estimates should be compared with caution, because age distributions of cattle and laboratory protocols differed among studies. One of the previous studies included only two large US herds [18], whereas the second study selected cattle only from test-positive dams [17]. Our results differed from those of Pithua et al. [19], who did not detect MAP culture-positive calves < three months of age, possibly because they used solid culture, which has lower sensitivity [30].

It is noteworthy, that one of the afore-mentioned studies also performed IS900 PCR [18]. Interestingly, their culture and IS900 prevalence were very similar to each other, in contrast to the present study where the IS900 prevalence was higher than the culture prevalence (8.1 versus 2.0%). The reason for this discrepancy was likely the serial testing scheme applied in the present study where only PCR-positive cattle were cultured which reduced the sensitivity of the testing scheme. This was supported by the fact that in the study mentioned before, different cattle tested positive on culture than on PCR [18]. Therefore, if they would only have cultured PCR-positives, their culture prevalence would likely also have been much lower, providing evidence for an underestimation of the prevalence of MAP shedding by culture estimates in our study. However, IS900 estimates have to be interpreted with caution because the IS900 element is also present in other bacteria, resulting in false-positive results [31]. A higher proportion of IS900 positives than F57 positives was expected, because IS900 is a multi-copy target and F57 is a single-copy target resulting in a lower detection limit for IS900 PCR [32].

Interpretation of prevalence estimates was aimed to reduce the number of misclassified cattle. We therefore performed three tests for prevalence estimation and interpreted results based on test combinations. The initial PCR screening was performed to identify samples that potentially contained MAP. Two PCR reactions with different primers were performed, which was a rapid and relatively inexpensive screening method ideal for processing many samples. Furthermore, this parallel testing resulted in higher sensitivity than sensitivities of the two separate tests [33]. This is supported by the fact that culture positive results were not only observed in young stock positive in both PCR methods, but also in young stock positive in only one of the two PCR methods. These shedders would have been missed if only one PCR method would have been conducted. Culture of any positives was done to increase specificity of the testing scheme. Culturing MAP is almost 100% specific [34], especially in the present study where cattle were unlikely to be housed in proximity to any high shedders or clinical cases of JD, thereby decreasing the probability of passive (pass-through) shedding. However, the prevalence estimated by the serial testing scheme is likely an underestimation of the true prevalence of shedding cattle, because some low shedders were likely missed.

Probability of shedding was associated with adult cow environmental culture prevalence, a proxy for within-herd MAP prevalence [28]. One obvious reason is that higher adult cow within-herd prevalence is associated with a higher infection risk and subsequently higher within-herd prevalence in young stock. A second reason would be that young stock are exposed to MAP more frequently and to higher doses if they are housed on high-prevalence farms, which would result in higher odds of shedding among infected cattle [16]. Therefore, shedding patterns in young stock on dairy farms with different within-herd prevalences should be investigated in a longitudinal study.

In the present study, MAP contamination was detected in 14% of calf and young stock group-housing pens, whereas 50% of farms had ≥ 1 environmental culture-positive pen. Previous work identified no positive pre-weaning calf pens and only 3% positive post-weaning calf pens [35]. Apparent discrepancies in results were attributed to the use of different culture protocols and differences in the study population (including uninfected herds in the previous study). It was noteworthy that environmental samples from pens with 6 months to 1 year-old young stock more frequently were culture-positive than environmental samples from pens with calves < 3 months. A possible explanation is the pen structure; young stock < 3 months were generally housed on straw packs without alleyways, which forced sample collection from bedding packs. In contrast, pens holding > 6 months old young stock usually had alleyways available for sample collection. Alleyway samples are more often culture-positive than bedding pack samples, perhaps due to increased mixing of manure in alleyway samples [36].

No MAP was isolated from any settled dust samples. A Dutch study used the same protocol and isolated MAP bacteria from young stock housings, but only if they were co-housed with cows [37]. However, in the present study, young stock and cows were usually housed in separate barns. It is therefore unlikely that infectious cows contaminated settled dust collected in this study. The amount of MAP excreted by infectious young stock might be too small to contaminate settled dust sufficiently to be detected with current culture methods and dust might be of minor importance for the transmission of MAP, as long as young stock and cows are housed independently.

Young stock > 6 months of age were not available for testing in one herd with serial testing scheme, impacting prevalence estimates to a limited extent. The prevalence of infectious cattle was low, thereby reducing the power for detecting associations between test results and independent variables. To mitigate this limitation, results and associations were described for all three test methods, making the assumption that misclassifications of cattle were predictor-independent in all tests (supported by descriptive statistics). Consequently, age and adult cow environmental sample results were significant predictors for IS900 results (13% prevalence), but did not predict F57 and culture results (~2% prevalence).

Samples were stored for a maximum of 21 days, which may have had a minor impact on the accuracy of the initial qPCR screening, since PCR does not require live bacteria. However, subsequent culture needed viable bacteria to become positive, suggesting an impact of sample storage conditions on accuracy of culture protocols in general. However, the thick cell wall of MAP enables it to survive in the environment for extended intervals [38-40]; it was estimated that MAP can be stored at 4 °C for at least 1 week without substantial loss in culture accuracy [41]. Therefore, we inferred that storage duration had only a minor impact on the sensitivity of MAP culture, although some samples with low bacterial concentrations were possibly misclassified as negative, which would have resulted in an underestimation of the prevalence of culture positive cattle and in an underestimation of the proportion of MAP contaminated pens.

Participating herds were more likely to have a history of observed clinical JD and were more likely to be culture-positive using environmental samples than non-participating herds. This was expected due to applied herd selection criteria. Therefore, results can be generalized to MAP environmental sample positive dairy farms with similar size and management.

This study provided clear evidence that naturally infected dairy calves can excrete MAP bacteria. Transmission of MAP between young stock was demonstrated previously [20], but the extent to which transmission events occur remains unknown. Consequently, a transmission trial is needed to quantify the potential for calf-to-calf transmission in group-housed dairy calves.

In conclusion, excretion of MAP by young stock occurred in MAP-infected dairy herds, with shedders present in all age groups. The odds of being IS900-positive was positively associated with prevalence of MAP-positive environmental samples of adult cattle housing and manure storage. Shedding of MAP lead to contaminated pens, especially in situations with a higher prevalence of MAP shedding cattle.

## Additional file

Additional file 1:
**Mycobacterium avium subspecies paratuberculosis (MAP) test results stratified by farm (# of positives (n tested)).** The file contains a Microsoft Word document with test results from all samples collected within the study. “Cow env. culture” refers to the results of environmental samples collected from adult cow housing and manure storage. “Environmental culture” refers to results of young stock environmental samples. “Individuals” refers to young stock fecal samples. These were processed using IS900 and F57 PCR. Any positives were subsequently cultured.

## Abbreviations

Ct: Cycle to positive
JD: Johne’s disease
MAP: *Mycobacterium avium* subspecies *paratuberculosis*
OR: Odds ratio
qPCR: Quantitative polymerase chain reaction
