# Supplementary material for: Calves shedding Mycobacterium avium subspecies paratuberculosis are common on infected dairy farms
Source: Vet Res. 2015 Jun 19;46(1):71. doi: 10.1186/s13567-015-0192-1 (PMC4474329; doi:10.1186/s13567-015-0192-1)
Supplement: Additional file 1: — Mycobacterium avium subspecies paratuberculosis (MAP) test results stratified by farm (# of positives (n tested)). The file contains a Microsoft Word document with test results from all samples collected within the study. “Cow env. culture” refers to the results of environmental samples collected from adult cow housing and manure storage. “Environmental culture” refers to results of young stock environmental samples. “Individuals” refers to young stock fecal samples. These were processed using IS900 and F57 PCR. Any positives were subsequently cultured. [file 13567_2015_192_MOESM1_ESM.docx]

**Additional file 1 *Mycobacterium avium* subspecies *paratuberculosis* (MAP) test results stratified by farm (# of positives (n tested)).**

|  | Cow env. culture^1^ |  | Individuals | | |  | Environmental |
| --- | --- | --- | --- | --- | --- | --- | --- |
| Herd |  |  | IS900 | F57 | Culture |  | culture |
| 1^2^ | 0 |  | 0 (34) | 0 (34) | 0 (0) |  | 0 (6) |
| 2 | 1 |  | 20 (178) | 0 (178) | 3 (49) |  | 0 (9) |
| 3 | 1 |  | 17 (109) | 20 (109) | 5 (43) |  | 1 (11) |
| 4 | 4 |  | 8 (153) | 2 (153) | 2 (12) |  | 1 (11) |
| 5 | 1 |  | 1 (104) | 0 (104) | 2 (4) |  | 3 (7) |
| 6 | 2 |  | 40 (130) | 0 (130) | 6 (45) |  | 4 (11) |
| 7 | 2 |  | 3 (121) | 0 (121) | 0 (4) |  | 0 (8) |
| 8 | 4 |  | 6 (227) | 1 (227) | 3 (16) |  | 1 (8) |
| 9 | 4 |  | 8 (154) | 0 (154) | 5 (11) |  | 2 (14) |
| 10 | 2 |  | 4 (135) | 1 (135) | 1 (6) |  | 2 (4) |
| 11 | 2 |  | 2 (76) | 0 (76) | 1 (3) |  | 0 (5) |
| 12 | 0 |  | 2 (162) | 0 (162) | 2 (4) |  | 0 (9) |
| 13 | 4 |  | 11 (158) | 0 (158) | 4 (12) |  | 0 (8) |
| 14^3^ | 3 |  | 19 (202) | 5 (202) | 5 (12) |  | 0 (3) |
| 15^3^ | 4 |  | 4 (114) | 0 (114) | 0 (7) |  | 0 (1) |
| 16^3^ | 3 |  | 23 (221) | 3 (221) | 2 (19) |  | 4 (11) |
| 17^3^ | 5 |  | 22 (214) | 0 (214) | 0 (5) |  | 0 (7) |
| 18^3^ | 4 |  | 20 (114) | 0 (114) | 1 (22) |  | 1 (6) |

^1^ Number of MAP environmental culture-positive samples out of 6 samples collected at the adult cows’ environment and manure storage.

^2^ Young stock > 6 months of age were on pasture and not available for sample collection.

^3^ Culture was conducted on a sub-set of PCR-positive samples.
